# Accelerating the design of lattice structures using machine learning

**DOI:** 10.1038/s41598-024-63204-7

**Published:** 2024-06-14

**Authors:** Aldair E. Gongora, Caleb Friedman, Deirdre K. Newton, Timothy D. Yee, Zachary Doorenbos, Brian Giera, Eric B. Duoss, Thomas Y.-J. Han, Kyle Sullivan, Jennifer N. Rodriguez

**Affiliations:** Lawrence Livermore National Laboratory, 7000 East Avenue, Livermore, CA 94550 USA

**Keywords:** Engineering, Mechanical engineering, Mechanical properties

## Abstract

Lattices remain an attractive class of structures due to their design versatility; however, rapidly designing lattice structures with tailored or optimal mechanical properties remains a significant challenge. With each added design variable, the design space quickly becomes intractable. To address this challenge, research efforts have sought to combine computational approaches with machine learning (ML)-based approaches to reduce the computational cost of the design process and accelerate mechanical design. While these efforts have made substantial progress, significant challenges remain in (1) building and interpreting the ML-based surrogate models and (2) iteratively and efficiently curating training datasets for optimization tasks. Here, we address the first challenge by combining ML-based surrogate modeling and Shapley additive explanation (SHAP) analysis to interpret the impact of each design variable. We find that our ML-based surrogate models achieve excellent prediction capabilities (*R*^2^ > 0.95) and SHAP values aid in uncovering design variables influencing performance. We address the second challenge by utilizing active learning-based methods, such as Bayesian optimization, to explore the design space and report a 5 × reduction in simulations relative to grid-based search. Collectively, these results underscore the value of building intelligent design systems that leverage ML-based methods for uncovering key design variables and accelerating design.

## Introduction

The design of architected structures and materials, often referred to as metamaterials, with tailored mechanical properties and functionalities remains a significant challenge due to the vast and complex parameter space^1–6^. With each added design variable, such as lattice type, shape, or size, the explorable parameter space rapidly becomes intractable and prohibits exhaustive brute-force searching to find optimal designs. Lattice structures, with features such as hierarchy patterning, internal structure, and architected designs, can exhibit diverse mechanical properties which are not typically achievable using designs available via conventional manufacturing. Examples of these properties are structures with low weight and high strength or having a negative Poisson’s ratio^7–11^. The intricacy resulting from these complex designs with diverse mechanical performance has motivated several research efforts to explore the use of computational approaches such as finite element analysis (FEA) to understand and explore the design space^12–14^. More recently, machine learning (ML)-based approaches have emerged as a feasible method to predict mechanical performance through building surrogate models to avoid the high computational costs of running FEA simulations^15–17^. Nevertheless, building these surrogate models requires the acquisition of sufficient data which can often be costly and laborious. Furthermore, surrogate modeling efforts often focus exclusively on prediction accuracy and neglect analyzing the effect of design variables on performance. While approaches such as active learning, where datasets are iteratively curated, have emerged to aid in rapidly navigating the parameter space, their application in the design of architected structures, such as lattice families, remains in its infancy^18–22^.

**Figure 1:**
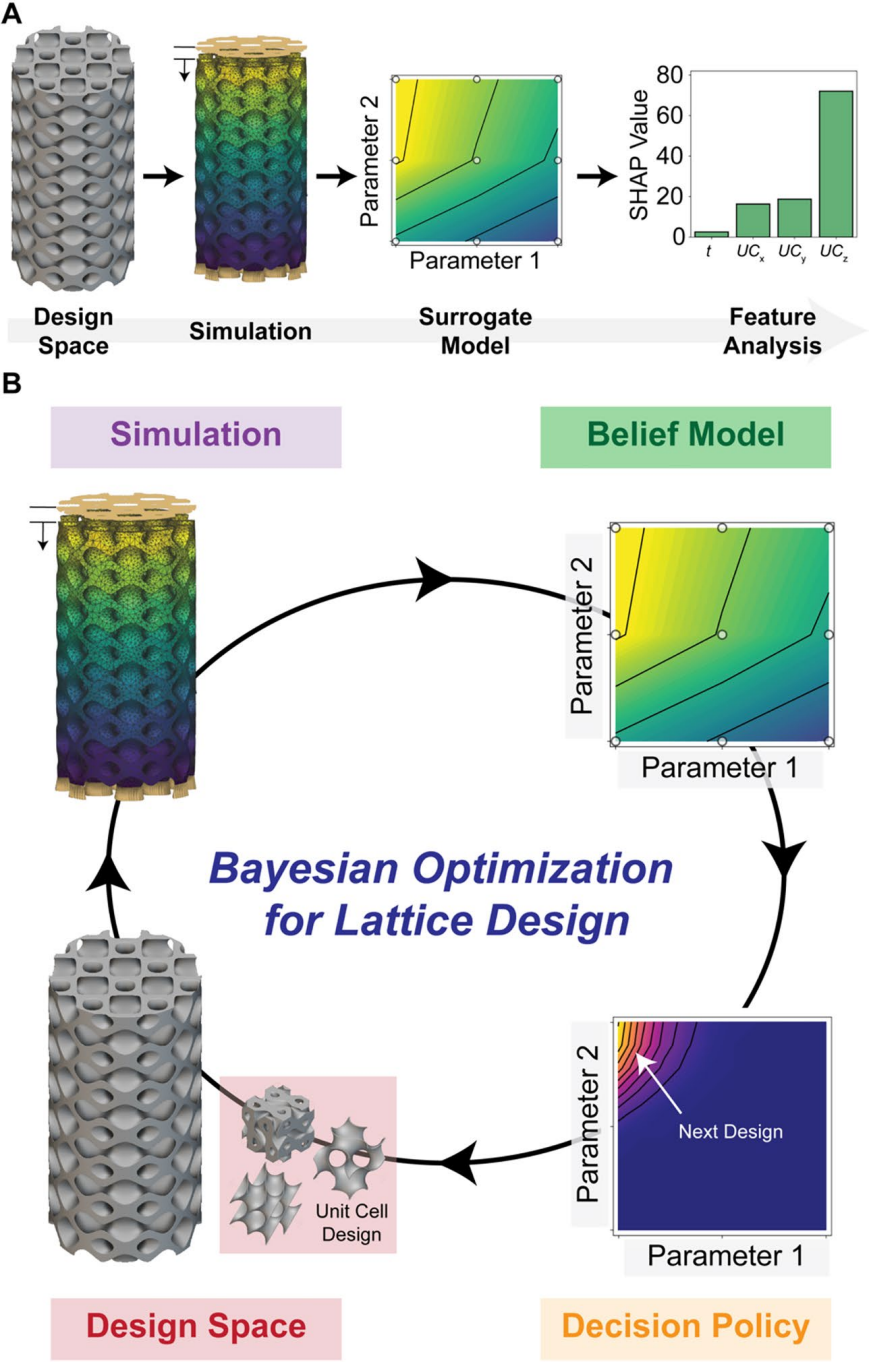
Machine Learning (ML) Framework to Interpret and Accelerate the Design of Architected Lattice Structures. (**A**) Pipeline for interpreting the impact of design variables on mechanical performance using simulation approaches such as finite element analysis (FEA) and interpretation techniques such as Shapley additive explanation (SHAP analysis. (**B**) Pipeline for the optimization of lattice structures where the design space is sequentially explored using a Bayesian optimization approach, where designs are selected and virtually tested to iteratively build a belief model and use a decision-policy to select the next simulation.

In this work, we demonstrate the use of ML-based approaches to address two key challenges: (1) building and interpreting ML-based surrogate models for mechanical performance and (2) iteratively and efficiently curating training datasets for optimizing mechanical performance. To address these challenges, we designed and utilized a framework that uses interpretable ML and surrogate modeling to reveal the impact of design variables on mechanical performance (Fig. 1A). Additionally, we designed and utilized a framework that uses Bayesian optimization to iteratively navigate the design space to rapidly identify high-performing lattice designs (Fig. 1B). For both applications, we generate a dataset for mechanical performance across five different lattice families and four geometric design variables, where we consider mechanical performance to be Young’s modulus $E$. The five lattice families correspond to lattice unit cell geometries, namely Schwarz, Lidinoid, Diamond, Gyroid, and Split P. The geometric design variables correspond to the size and thickness of the lattice unit cell. We use the framework to address the first challenge by combining ML-based surrogate modeling and Shapley additive explanation (SHAP) analysis to interpret the impact of each design variable. We find that our ML-based surrogate models achieve excellent prediction capabilities (*R*^2^ > 0.95) and SHAP values aid in uncovering design variables influencing performance. We observe that design variables such as the size of the unit cell in the direction of the applied load has the largest effect on $E$. Next, we use the framework to address the second challenge by utilizing active learning-based methods, such as Bayesian optimization (BO), to explore the design space. We find that our custom active learning approach can find optimum structures with 82% less simulations relative to grid-based search for a performance target greater than or equal to 0.90. Collectively, these results underscore the value of leveraging ML-based methods for uncovering key design variables and accelerating design.

## Results and discussion

### The parametric design space

Architected materials are engineered to possess desired performance by carefully controlling the arrangement of material and geometry. Due to the range of diverse behaviors achieved by architected materials, exciting work has emerged, from discovering and harnessing design principles to rationally exploring new functionalities, properties, and applications. In the design of mechanical metamaterials, research has demonstrated the design of lightweight materials with negative Poisson’s ratios and programmable mechanical responses^7,10^. In addition to the field of mechanics, architected materials have gained popularity in optics^23,24^, acoustics^25,26^, and thermal applications^27,28^. While these studies have demonstrated the benefit of embracing a new design paradigm based on leveraging architecture, morphology, and hierarchy to continually develop mechanical and physical properties, a major challenge to the widespread adoption and use of lattice structures is being able to rapidly and intelligently select the lattice design to meet application requirements. Consequently, achieving target performance and functionality will require utilizing approaches that enable the swift exploration of the material-architecture-property design space to find optimal designs.

**Figure 2:**
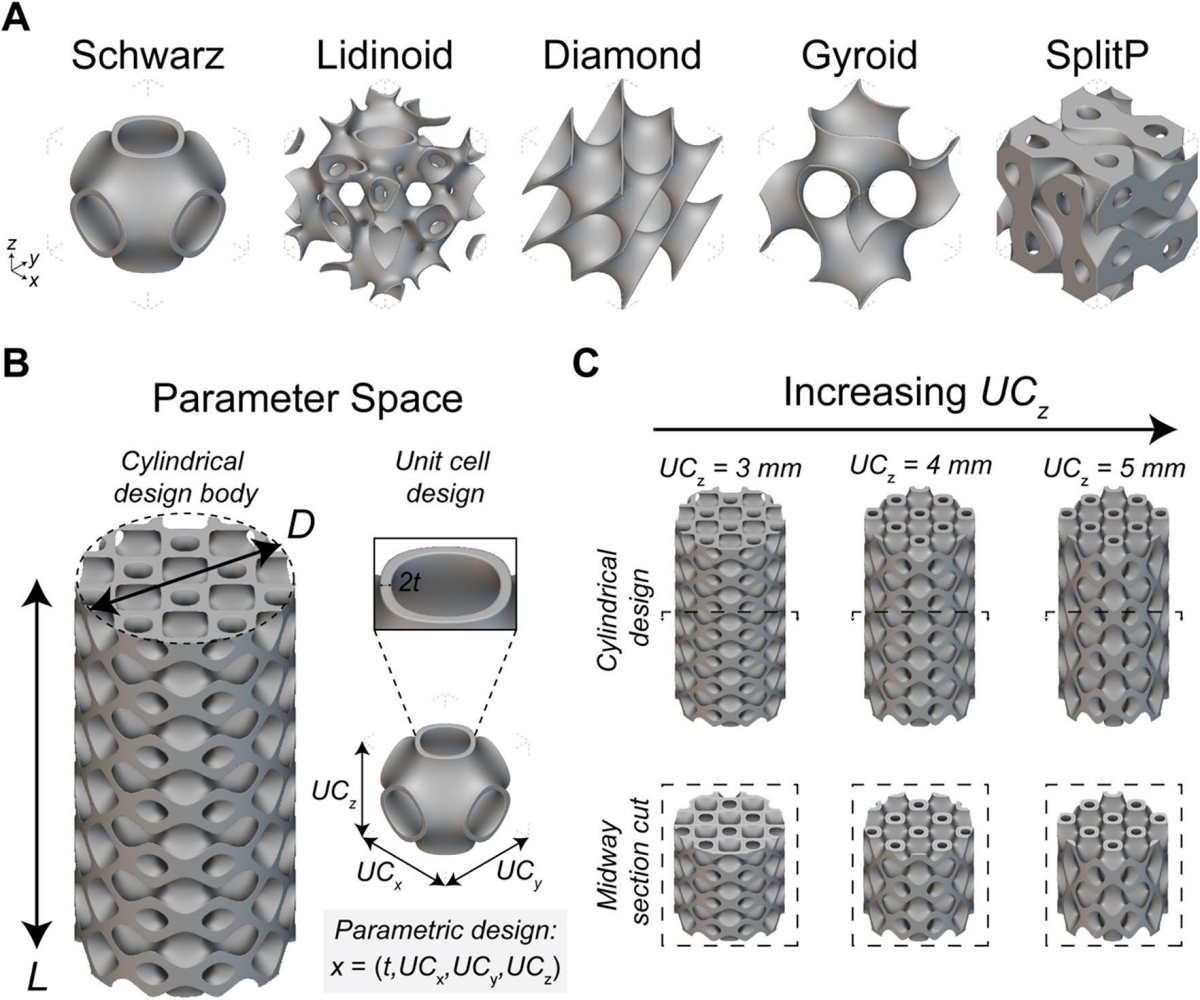
The Parametric Lattice Parameter Space. (**A**) The five selected triply periodic minimal surface (TPMS) unit cells were Schwarz, Lidinoid, Diamond, Gyroid, and Split P. (**B**) The parametric space of unit cells patterned on a cylindrical design body with diameter $D$ and length $L$ and parametrized as $x=\left(t, U{C}_{x}, U{C}_{y}, U{C}_{z}\right)$. (**C**) An illustration of how changes in $U{C}_{\text{z}}$ changes the lattice architecture.

In this work, we address this challenge by developing an ML-based framework for understanding and accelerating the design of architected lattices. The ML-based framework builds on interpretable ML and active learning approaches to navigate the mechanical performance design space. Here, we focus on triply periodic minimal surface (TPMS) designs since prior work has shown that they exhibit superior mechanical properties relative to other lattice types^13,29^. TPMS designs seek to minimize the surface area for a given boundary where the mean curvature of each point on the surface is zero. TPMS designs feature interesting geometric characteristics, such as no sharp edges or corners, and divide the space into nonintersecting domains that can be periodically repeated in three perpendicular directions. Here, we construct a parametric design space using five selected TPMS unit cells, namely Schwarz, Lidinoid, Diamond, Gyroid, and Split P (Fig. 2A). The TPMS unit cells are then mapped on a cylindrical design body with length $L$ = 20 mm and diameter $D$ = 10 mm (Fig. 2B). The unit cell design is also further parametrized by design variables such as the thickness $t$ and size of the unit cell $U{C}_{x}$, $U{C}_{y}$, and $U{C}_{z}$, which correspond to the length of the unit cell in the $x$, $y$, and $z$, directions, respectively (Fig. 2B).

For each TPMS unit cell design, the design variables can be parametrized as ${x}_{UC}=\left(t, U{C}_{x}, U{C}_{y}, U{C}_{z}\right)$. With this parametrization, we can observe changes in the physical properties. For example, with the Schwarz cell, we can see the changes in the geometry as the size of the unit cell increases in the z-direction (Fig. 2C). The volume fraction of the structure decreases from 0.35 to 0.32 and finally to 0.29 when $U{C}_{z}$ ranges from 3, 4, and 5 mm. To investigate the mechanical performance of various lattices families as a function of geometric changes, we developed a custom automated finite element analysis (FEA) pipeline to predict the effective Young’s modulus $E$ of the designs and to calculate $\widetilde{E}$ as the ratio between $E$ and the mass $m$ of the design (Figure S1).

Both $E$ and $\widetilde{E}$ are important mechanical parameters because they both describe the elastic behavior of a structure under an applied force, which is critical to design applications. The Young’s modulus represents the elastic response of homogenous isotropic materials, which describe the elastic behavior of a structure under an applied force. The Young’s modulus can be measured as the slope of the initial elastic linear region in the stress–strain curve. Due to the influence of geometry on the stress–strain response, we refer to the slope of the initial elastic linear region as the effective Young’s modulus $E$ since it captures the effects of both the material and the geometry. Additionally, we define $\widetilde{E}$ as the ratio between $E$ and the mass $m$ of the design which is calculated as $\widetilde{E}= \frac{E}{m}$ . Here, $m$ is calculated as $m=\rho V$ where $\rho = 1.25 g/c{m}^{3}$, which is the density of PLA, and $V$ is the volume of the design. From the parametric designs, we convert the designs to a tetrahedral mesh and then simulate a uniaxial quasi-static compression test by displacing the nodes on the top surface while fixing the nodes on the bottom surface using commercial software (nTopology). From the FEA simulation, we can calculate

$$E = \frac{FL}{{A\Delta z}}$$

where

$$F=reaction\; forces$$

$$L=design\; height$$

$$A=area\; of\; the\; top\; surface$$

$$\Delta z=prescribed\; displacement\; of\; the\; top\; nodes.$$

Interestingly, we observe a corresponding increase in the Young’s modulus $E$ from 156 to 293 MPa and finally to 358 MPa with increasing $U{C}_{z}$. The observed 2.3 × increase in $E$ with a 17% decrease in volume fraction is a favorable trend in mechanical properties, as it indicates an increase in mechanical performance with a potential decrease in material needed, highlighting the influence of geometry on performance.

## Investigating the mechanical performance via surrogate modeling

To investigate the mechanical performance in the parameter space, we employ a grid-based search approach and divide the design variables for each individual lattice design, with $t$ being 0.3 and 0.4 mm and $U{C}_{x}$, $U{C}_{y}$, and $U{C}_{z}$ being 3, 4, and 5 mm each. With this method, each lattice design is sampled at 54 unique locations within the parameter space, resulting in a total of 270 unique locations across all five lattice designs investigated. The number of points is selected to balance computational cost and exploration of the parameter space. Using the selected 270 unique locations, the FEA pipeline was used to estimate the Young’s modulus $E$ and the ratio $\widetilde{E}$ for all the designs.

**Figure 3:**
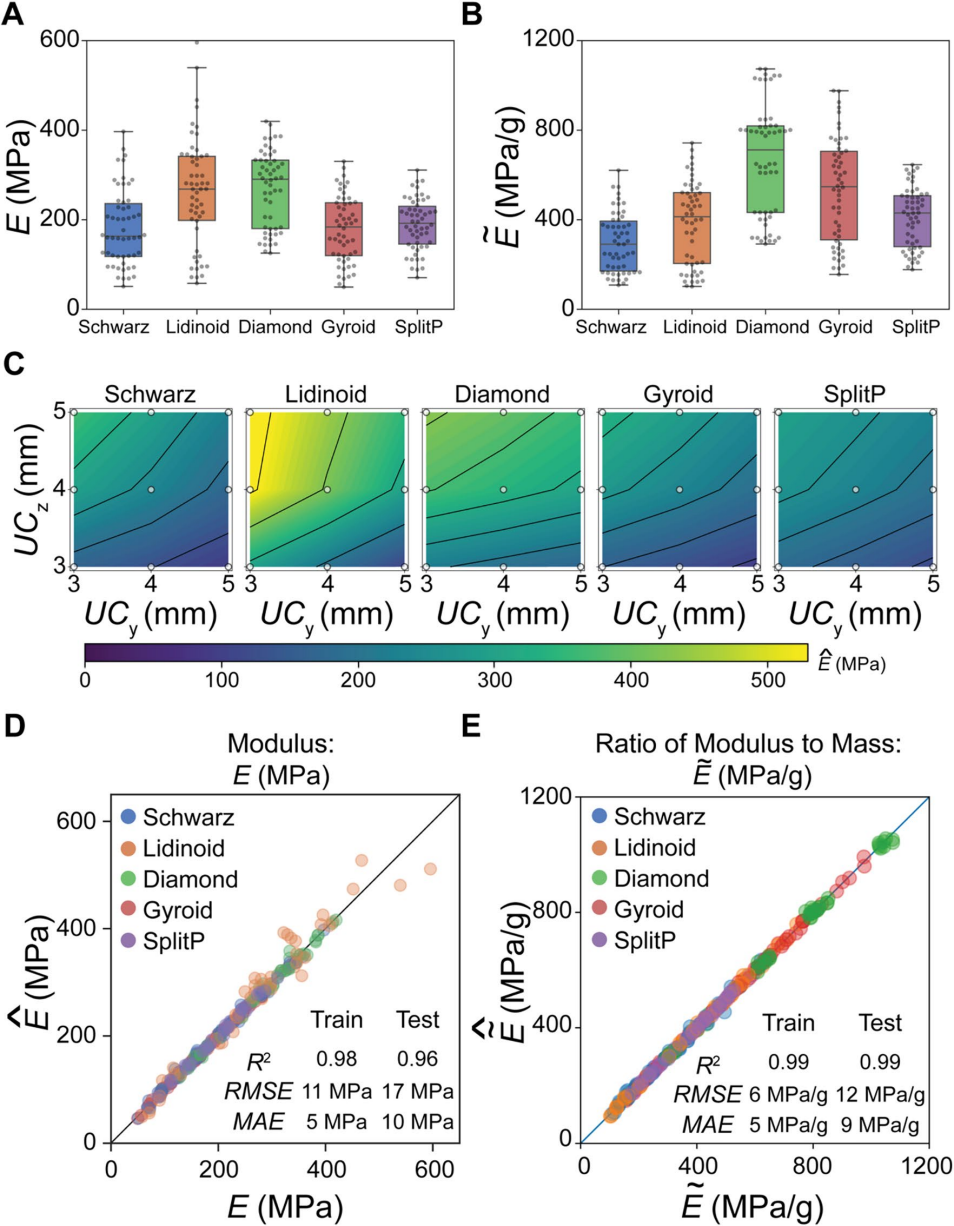
Grid-based Search to Build Machine Learning (ML)-based Surrogate Models to Explore the Lattice Design Space. Box and whisker plots for (**A**) Young’s modulus $E$ and (**B**) ratio of Young’s modulus to mass $\widetilde{E}$. (**C**) Machine learning (ML)-based surrogate model predictions of $E$ as a function of $U{C}_{y}$ and $U{C}_{z}$ where $t$ = 0.3 mm and $U{C}_{x}$ = 3 mm. (**D**) Parity plot of predicted modulus $\widehat{E}$ versus observed modulus $E$ for both testing and training data. (**E**) Parity plot of predicted values $\widehat{\widetilde{E}}$ versus observed values $\widetilde{E}$ for both testing and training data .

From the collected dataset of mechanical performance, the mean $E$ was 217 MPa with a standard deviation of 96 MPa while the mean $\widetilde{E}$ was 460 MPa/g with a standard deviation of 231 MPa/g. The maximum $E$ was 600 MPa, which corresponded to the Lidinoid lattice, while the minimum $E$ was 50 MPa, which corresponded to the Gyroid lattice (Fig. 3A). However, the maximum $\widetilde{E}$ was 1074 MPa/g, which corresponded to the Diamond lattice, while the minimum $\widetilde{E}$ was 102 MPa/g, which corresponded to the Lidinoid lattice (Fig. 3B). In both $E$ and $\widetilde{E}$, an order of magnitude difference between the lowest and highest performing designs was observed, demonstrating the wide range of mechanical performance achieved by the TPMS design family. Interestingly, based on average performance, the mean $E$ and $\widetilde{E}$ of the diamond lattice design was the largest.

To better explore the mechanical performance of the lattice designs throughout the parameter space, we employed surrogate modeling methods to approximate the design space, using the collected dataset. For example, we can visualize a slice of the parameter space near the optimum observed $E$ = 600 MPa where ${x}_{UC}=\left(t=0.3, U{C}_{x}=3, U{C}_{y}=3, U{C}_{z}=5\right)$ mm. By keeping $t$ = 0.3 mm and $U{C}_{x}$ = 3 mm constant, we can observe $E$ as a function of $U{C}_{y}$ and $U{C}_{z}$ (Fig. 3C). In this vicinity, we observe that E increases as $U{C}_{y}$ decreases and $U{C}_{z}$ increases for all five lattice designs. Interestingly, we also observe that *E* decreases as $U{C}_{y}$ increases and $U{C}_{z}$ decreases for all give lattice designs.

The surrogate model used to explore the parameter space of $E$ was a Gaussian process (GP) model with automatic relevance determination (ARD), built using a 75/25 training/testing split of the dataset. From the parity plot of predicted Young’s modulus $\widehat{E}$ versus $E$, the model was observed to possess excellent prediction capabilities with a Pearson correlation coefficient (${R}^{2}$) at 0.96, a root mean square error (RMSE) at 17 MPa, and a mean absolute error (MAE) at 10 MPa for the testing dataset (Fig. 3D). Additionally, we built a surrogate model for $\widetilde{E}$ using a similar approach. From the parity of predicted values $\widehat{\widetilde{E}}$ versus $\widetilde{E}$, the model was observed to also possess excellent prediction capabilities with ${R}^{2}$ = 0.99, RMSE = 12 MPa/g, and MAE = 9 MPa/g for the testing set. (Fig. 3E). While both surrogate models offer excellent prediction capabilities, we also note that the predictions are not perfect. We observe that the maximum relative error observed in the predictions of the surrogate model for $E(x)$ was 20%, while ~ 84% of predictions possessed $\le$ 5% relative error. We also observe that the maximum relative error observed in the predictions of surrogate model for $\widetilde{E}\left(x\right)$ was 14%, while 85% of predictions possessed $\le$ 5% relative error.

While the surrogate model for $E(x)$ was determined to be satisfactory for prediction tasks, we also observe discrepancies at higher $E$ values for the Lidinoid structures. We surmise that this discrepancy stems from the data sparsity at these locations arising from the grid-based sampling approach used to explore the design space for the five lattice designs. This is observed in the histogram of $E$ (Figure S7) where majority of data points are located below 400 MPa. Additionally, we observe that in cases when there is a larger data density (Figure S8), such as in $\widetilde{E}\left(x\right)$, we observe better prediction capabilities. While rigorous analysis of the effect of data density and sampling on surrogate model building is outside the scope of this work, we highlight relevant observations for designers and researchers that are interested in using surrogate modeling approaches in their workflow.

Firstly, we note that in the presence of data sparsity, the surrogate models may still be able to (i) correctly identify regions of high performance in the design space at the expense of prediction fidelity and (ii) decrease prediction after training the surrogate model. In this study, we observe that the surrogate model for $E(x)$ possesses larger discrepancies for pointwise predictions of $E(x)$ for Lidinoid designs but can still correctly identify that the Lidinoid designs are high performing. Additionally, we also observe a significant decrease in prediction time where the surrogate model can predict $E(x)$ values in less than 1 s, as opposed to the approximate computational cost of 37 min per simulation (Figure S6). We highlight these insights to provide designers and researchers with guidance when considering using surrogate modeling approaches in their workflows and emphasize the potential trade-offs in prediction fidelity and prediction time.

**Figure 4:**
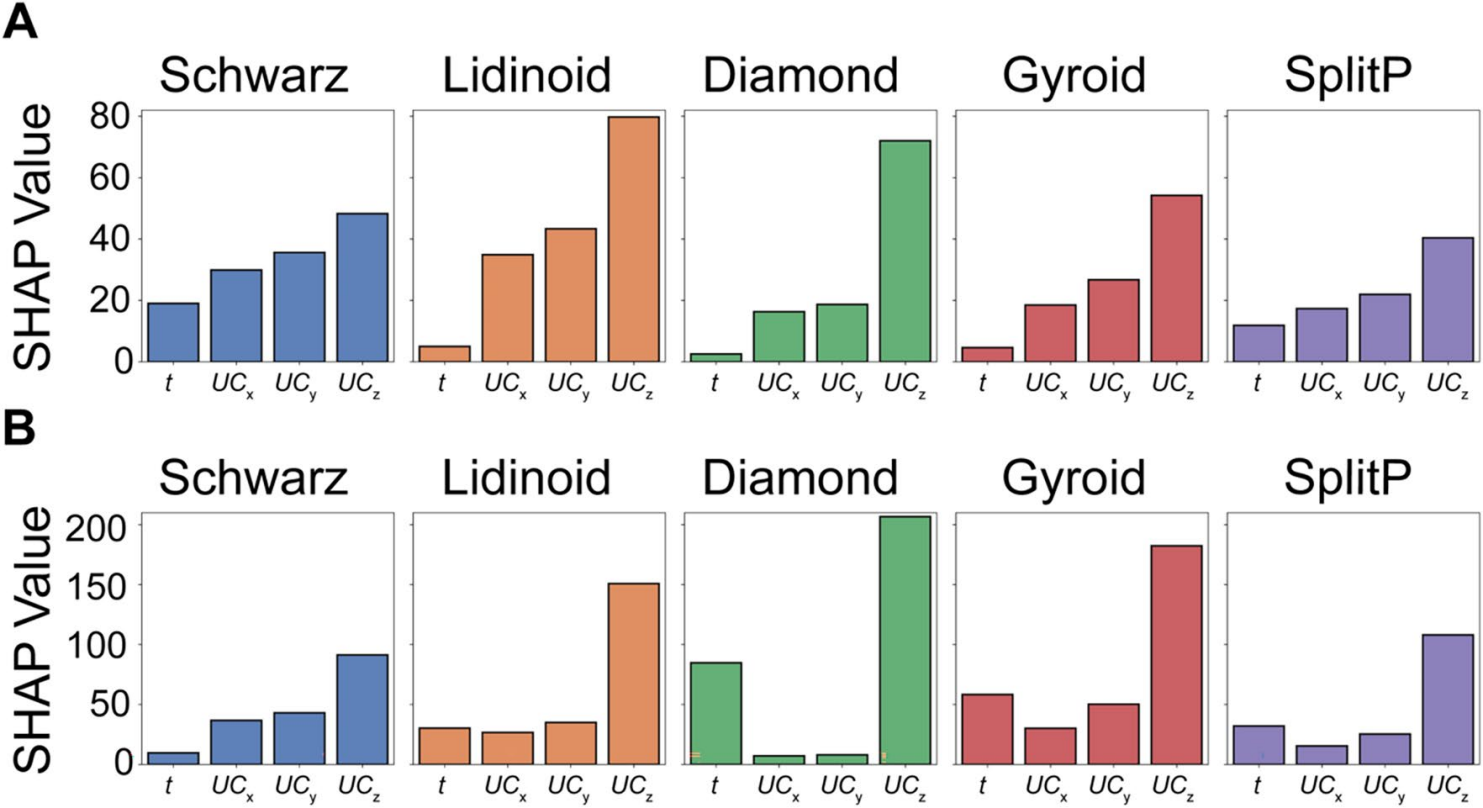
Global Interpretability of the Machine Learning (ML)-based Surrogate Models using Shapley Additive Explanations (SHAP). The mean absolute value of each design feature over all instances in the training set for (**A**) $E(x)$ and (**B**) $\widetilde{E}\left(x\right)$.

Given the excellent prediction capabilities of the ML-based surrogate models and the reduced computational expense, we used these surrogate models as ground truth representations of the parameter space and refer to them as $E(x)$ for Young’s modulus and $\widetilde{E}\left(x\right)$ for the ratio of Young’s modulus to mass. With these models, we utilized Shapley Additive Explanations (SHAP) for global interpretation of their predictions. SHAP is a useful approach in interpreting the output of an ML model by decomposing the prediction of an ML model into a sum of the contributions from each design variable. Using the SHAP values, we can investigate and better understand the impact of the design variables on $E(x)$ and $\widetilde{E}\left(x\right)$. Based on the mean average SHAP values for $E(x)$, we observe that $U{C}_{z}$ has the largest influence on $E(x)$ while $t$ has the least influence for all the lattice designs. Of all the lattices, $U{C}_{z}$ has the greatest influence on the predictions of the Lidinoid designs, which was also the design with the optimum $E$ (Fig. 4A). Based on the SHAP values for $\widetilde{E}\left(x\right)$, we also observe that ${UC}_{z}$ has the largest influence for all the lattice designs. ${UC}_{x}$ and ${UC}_{y}$ have similar influences, which may be due to the symmetry of the cylindrical design body. However, for the Schwarz and Lidinoid designs, the second most influential design variable is ${UC}_{y}$ while for the Diamond, Gyroid, and Split P designs, the second most influential design variable is $t$ (Fig. 4B). These observations from SHAP analysis represent a means of understanding the effect of the design variables on $E(x)$ and $\widetilde{E}\left(x\right)$.

While the importance of the design features on the prediction of $E$ and $\widetilde{E}$ can be inferred from the mean absolute SHAP values, the distribution of all SHAP values for each feature is also informative. The distribution of SHAP values can be visualized using swarm plots, where the design variables are arranged from top to bottom in order of decreasing influence on the model predictions, namely on $E(x)$ (Figure S2) and $\widetilde{E}\left(x\right)$ (Figure S3). Each circular marker in the swarm plot signifies an entry in the dataset, where blue and red markers indicate lower and higher values of the feature, respectively. For example, blue markers in the ${UC}_{z}$ row represent lower values of ${UC}_{z}$ while red markers represent higher values of ${UC}_{z}$ (Figure S2). On the x-axis, the distance between a marker and the vertical line indicates the SHAP value for the individual entry. Together, the swarm plot can be used to understand the distribution of SHAP values and their respective influence on the model. In the case of ${UC}_{z}$, we can infer the lower values of ${UC}_{z}$ (blue markers) result in a strong negative contribution to $E(x)$ while higher values of ${UC}_{z}$ (red markers) result in a strong positive contribution to $E(x)$ . This indicates that the ML-based model has captured the ${UC}_{z}$ has the most significant influence on both $E(x)$ and $\widetilde{E}\left(x\right)$. One must note that SHAP values only capture the effect of the individual design variables and not potential coupled effects between parameters. In this work, we highlighted the use of SHAP for feature importance. While approaches such as SHAP quantify the contribution of each design variable to the performance, we would also like to highlight that these approaches can be limited when the design space becomes too large. In these cases, unsupervised feature reduction and selection approaches such as Principal Component Analysis (PCA) can be used in tandem with SHAP analysis^30,31^.

## Bayesian optimization for single lattice designs

While the previous grid-based search approach allowed for building an accurate ML-based surrogate model to identify high-performing lattice designs such as the Lidinoid lattice family, the number of simulations required to train the surrogate model can be prohibitively expensive. Here, the 270 simulations conducted using a design of experiments (DOE) approach were a minor fraction (< 1%) of the theoretical designs in the parameter space. Assuming a resolution of 25 microns in the $x$, $y$, and $z$ directions, the number of uniquely printable designs is > 10 million for the defined parameter space, which could rapidly be expanded to include other lattice families or geometric variables. To this end, exhaustively exploring the parameter space is impractical and requires a more robust and efficient means of navigating the design space that go beyond purely DOE approaches.

Fortunately, recent works have demonstrated the success of combining DOE approaches with active learning approaches such as Bayesian Optimization (BO)^32,33^. Active learning approaches provide a means of intelligently and iteratively selecting subsequent designs to meet a pre-defined goal. In particular, Bayesian optimization focuses on intelligently selecting subsequent queries to find optimal conditions in the least number of iterative steps. Unfortunately, these approaches have not been widely employed in the design of lattice structures. By integrating and leveraging techniques in active learning, the computational and experimental cost of lattice design could be drastically reduced by intelligently selecting designs or families within the vast parameter space.

In this work, we leverage the standard Bayesian optimization method where the Gaussian process (GP) is used as the surrogate model that the acquisition function uses to select the next design. After the selected design has been evaluated, the GP is retrained using the newly acquired data points. To avoid conducting the full-scale FEA simulations in each query of the BO agent, we use a separate ground truth surrogate model in lieu of the FEA simulator. The BO agent can only query the ground truth surrogate model at each iteration and has no additional available information. The advantage of using a ground truth surrogate model in lieu of the FEA simulator is that we can evaluate the performance of the BO agent, which would be otherwise be computationally infeasible given the total number of simulations conducted. Additionally, the use of surrogate models in the BO loop to assess performance has also been previously used and reported^34^.

**Figure 5:**
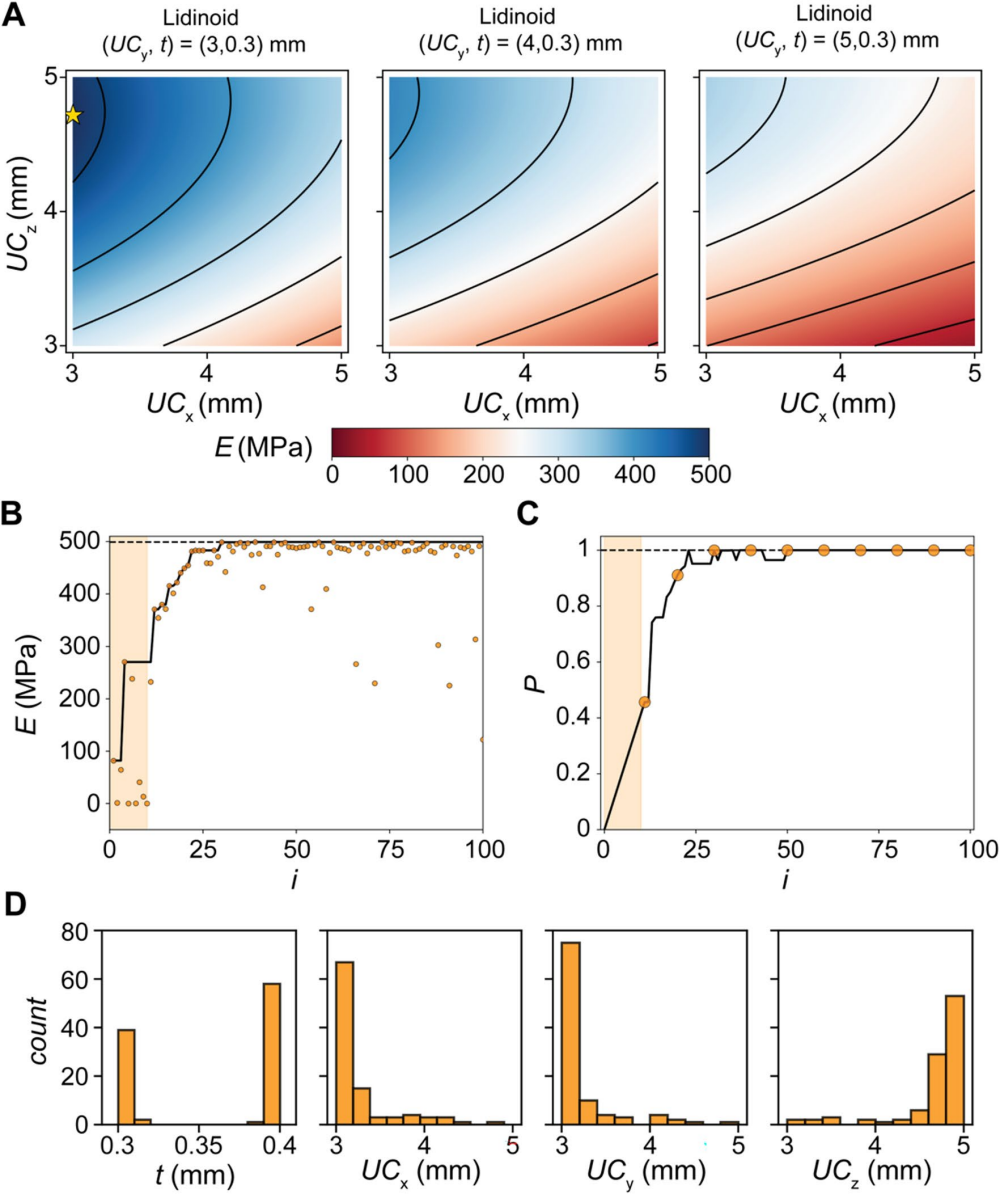
Independent Single Agent Bayesian Optimization (BO) Campaigns to Identify Optimum $E$ for the Lidinoid lattice family. (**A**) Contours across the parameter space for the Lidinoid design family to demonstrate the location of the optimum design. (**B**) Results from the independent BO where the $E$ from iteratively selected designs is plotted against the number of simulations $i$. (**C**) Results from the independent BO where the performance $P$ of iteratively selected designs is plotted against the number of simulations $i$. (**D**) Histograms of each design variable at the end of the independent campaign.

To explore and quantify the potential acceleration of active learning approaches, we developed and utilized a framework based on DOE and Bayesian optimization (Fig. 1) to identify high-performing designs. First, we used the surrogate model $E(x)$ to identify the lattice family that possessed the lattice design with the largest $E$, which was the Lidinoid design. Next, we identified the design parameters that corresponded to the top performing Lidinoid design ${x}^{*}=\left(t, U{C}_{x}, U{C}_{y}, U{C}_{z}, \right)=\left(0.3, 3, 3, 4.7\right) \text{mm}$ (Fig. 5A), which is labeled using a star marker in the contour map. Interestingly, we observe similar trends across the three slices where $E$ decreases as $U{C}_{x}$ increases and $U{C}_{z}$ decreases. Using the framework, we employed an independent single-agent BO campaign to identify high-performing structures in the Lidinoid design family with a simulation budget of 100. To initialize the campaign, 10% of the simulation budget was used to warm start the search using a Latin Hypercube sampling (LHS) approach. A gaussian process with automatic relevance determination was used as the belief model and an expected improvement (EI) decision policy was employed.

From the iterative selections of the BO agent, we observe convergence on high-performing designs after ~ 25 simulations (Fig. 5B). An interesting feature of the BO approach observed in the independent campaign is the balance between exploration and exploitation because of the selected EI policy. While design selections in the latter half of the campaign were primarily high-performing as a result of the exploitative nature of the EI policy, low-performing designs were sampled as a result of the exploratory nature of the EI policy. To better quantify the performance of the BO agent, we defined performance $P$ as $\frac{E\left(\text{argmax}\left({B}_{i}\left(x\right)\right)\right)}{\text{max}\left(E\left(x\right)\right)}$ , where ${B}_{i}\left(x\right)$ is the belief model trained after $i$ simulations. With this definition, $P=1$ indicates that the theoretical maximum has been found and values close to $P=1$ indicate high-performing designs. Using this metric, the performance of the BO agent can be observed to converge steadily after 28 simulations (Fig. 5C). Furthermore, we note that the BO agent primarily selects designs near the optimum ${x}^{*}$, indicating high density sampling close to the theoretical maximum (Fig. 5D).

As a single independent campaign provided valuable information about the approach, we proceeded to conduct 50 independent campaigns for each of the 5 lattice families (Figure S3). To assess the acceleration performance of the BO agent in finding optimal designs, we defined set a target threshold of $P\ge 0.95$. From the simulated learning campaigns, we observe that the Gyroid lattice design family required the least number of simulations (20) while the Lidinoid design family required the most (28 simulations). Considering the deployment of 5 independent BO agents across the 5 designs, the total number of simulations required to find the optimal design lattices was 156. These results suggest that 5 independent BO agents searching across the lattice design space would require 42% less simulations relative to a grid-based search. These results demonstrate the achievable acceleration enabled by employing active learning methods such as BO in lattice design.

## Bayesian optimization to explore the family of lattice designs

While deploying multiple independent campaigns resulted in a 42% reduction in simulations relative to grid-search, the campaigns were conducted individually for each lattice family. An open question that emerged was how to optimize simultaneously over the mixed-search space of the five lattice families. Here, a mixed-search space refers to the presence of both discrete and continuous design variables. In real applications, discrete variables can be the type or composition of material selected, while continuous design variables can be the amount of material or the size of the component. In our case, the independent design families are the discrete design variables while the dimensions of the unit cell and wall thickness are continuous design variables. Interestingly, despite the prevalence of optimized problems defined over mixed search spaces, to the optimal approach remains an on-going open question^35–37^.

To explore whether greater acceleration can be achieved by employing a mixed-search space BO strategy, we designed a set of simulated learning campaigns that combine our BO approach with one-hot encoding. One-hot encoding is an approach that assigns an integer representation for each category where no ordinal relationship exists between them and the similarity between any two categories is assumed to be equal^38,39^. Given these considerations and keeping the independence and similarity between the lattice design families, we selected one-hot encoding as a method to reparametrize our feature space (Figure S5). For this reparameterization, our design space is redefined as ${x}_{UC}=\left({x}_{OH},t, U{C}_{x}, U{C}_{y}, U{C}_{z}\right)$, where ${x}_{OH}$ is the one-hot encoded vector that corresponds to each lattice design. For example, the Lidinoid lattice design corresponds to ${x}_{OH}=\left(\text{0,1},\text{0,0},0\right)$.

**Figure 6:**
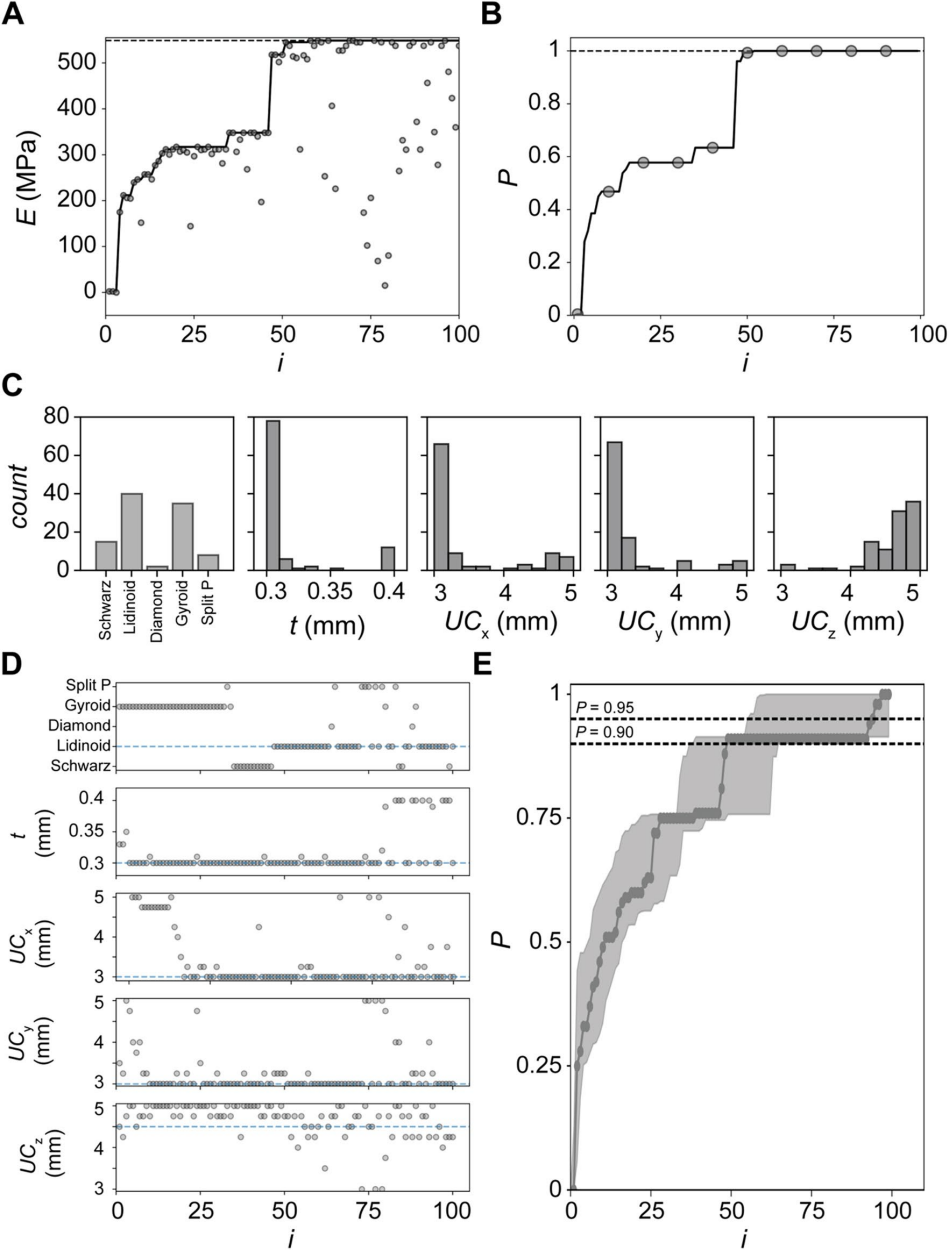
Bayesian Optimization (BO) Campaigns to Identify Optimum $E$ Across All Lattice Design Families. (**A**) Results from BO combined with one-hot encoding to iteratively select designs in a mixed search space where the $E$ of iteratively selected designs is plotted against the number of simulations defined as $i$. (**B**) Results from BO combined with one-hot encoding to iteratively select designs in a mixed search space where the performance $P$ of iteratively selected designs is plotted against the number of simulations defined as $i$. (**C**) Histograms of each design variable at the end of the independent campaign. (**D**) Scatter plots of each design variable selected along the trajectory of the campaign. (**E**) The performance $P$ of the BO agent versus the simulation number for 50 independent simulations where the markers correspond to the median performance and the shaded area correspond to the 25th and 75th quantiles.

With the reparameterization of the lattice design space, we conducted BO campaigns in the mixed search space. The budget of 100 simulations was kept constant and an identical belief model and acquisition function were used. For these campaigns, we did not warm start the campaign using LHS, as we did not want to bias the search by including a selection from the discrete design families in the initialization pool. The independent campaigns were seeded with a random design to start the campaign. From a single independent campaign, we observe convergence on the optimum design after 50 simulations (Fig. 6A). Using the metric for performance $P$, we also observe convergence to $P=1$ after 50 simulations (Fig. 6B). In the single independent campaigns, we also observe the balance between exploration and exploitation towards the end of the campaign where general convergence is observed, but the BO agent infrequently selects lower performing designs. Due to the balance between exploration and exploitation in the EI decision-policy, the BO agent can successfully identify the local maximum and avoid stagnation.

An additional interesting aspect is the distribution of design variables at the end of the campaign (Fig. 6C). After 100 simulations were selected by the BO agent, we observe that two lattices were primarily selected, namely the Gyroid and Lidinoid designs. The selection of the Gyroid designs corresponds to the initial plateau observed in the campaign (Fig. 6D). The distribution of the parameters and the sequential design choices provide insight into the BO agent’s balance between exploration and exploitation, where we observe faster convergence on design variables $t$ and $U{C}_{z}$ relative to $U{C}_{x}$ and $U{C}_{y}$, which had a greater variance in the selections earlier in the campaign. To assess the performance of the BO agent, we conducted 50 independent campaigns with a target threshold performance of $P\ge 0.90$ and $P\ge 0.95$ (Fig. 6E). We observe convergence after 50 simulations for $P\ge 0.90$, which corresponds to an 81% reduction in the number of simulations relative to grid-based search. Notably, the 81% reduction in the number of simulations demonstrates a significant ~ 5 × reduction in the empirical load in identifying high-performing designs. However, when a performance target of $P\ge 0.95$ is employed, convergence if observe after 94 experiments which corresponds to a 65% reduction in the number of simulations. In both cases, these results represent superior performance relative to individual BO campaigns. An additional highlight is the reduction in the computational load from approximately 7 days to 1.5 days which is approximated using an average run time of 37 min per simulation for a $P\ge 0.90$ or a reduction in the computational load from 7 days to 2.4 days for a $P\ge 0.95$. The average run time was approximated using the average computational run time from the grid-based search which is reduced from approximately 7 days (or 270 simulations) to 1.3 days (50 simulations) or 2.4 days (94 simulations). This is approximated using an average run time of 37 min per simulation.

These results indicate the versatility of employing active learning approaches such as Bayesian optimization in both continuous and mixed search spaces. In this work, we have demonstrated the use of BO for rapidly finding structures with maximum $E$ or $\widetilde{E}$. While not explicitly demonstrated in this work, we would also like to highlight that this method can be used for the inverse design of arbitrary values of $E$ or $\widetilde{E}$ by reformulating the objective function. Traditionally, the objective function in a BO approach is formulated as ${x}^{*}=\text{argmax}\left(E\left(x\right)\right)$ where ${x}^{*}$ corresponds to the design with the maximum $E$. To use the BO approach for the inverse design of arbitrary target values, the objective function could be expressed as ${x}^{*}=\text{argmin}\left({\left(E\left(x\right)-{E}_{Target}\right)}^{2}\right)$ where ${E}_{Target}$ is the target effective modulus. In this formulation, the goal of the BO agent is expressed as a minimization problem and the square of the error is used to make the objective function positive definite.

Furthermore, we highlight the use of the surrogate models to assess the performance of BO across several independent simulated learning campaigns due to the computational speed-up in using the surrogate model. We assessed performance across 50 independent simulated learning campaigns for each described approach, which includes campaigns for each lattice design and a combination of traditional BO with one-hot encoding. Due to the computational cost of directly running the FEA simulator in the BO loop for this many cases, the use of the surrogate model was deemed as an appropriate alternative. Furthermore, the use of surrogate models in the loop to assess BO performance has also been previously reported^34^.

## Conclusions

In this work, we demonstrate an approach to accelerate the design of lattice structures for mechanical performance by combining finite element analysis (FEA) and machine learning (ML). We first demonstrate the utility of using ML-based surrogate modeling and Shapley additive explanation (SHAP) analysis to interpret the impact of design variables on mechanical performance such as Young’s modulus $E$. This approach highlighted the impact of the unit cell in the direction of loading, with a longer unit cell in the direction of loading resulting in superior performance. Notably, this approach demonstrates the advantage of combining ML-based surrogate modeling and explainable A.I. to reveal the role of design variables in regimes of data paucity since < 1% of all the theoretical unique designs were needed to build a surrogate model with ${R}^{2}>0.95$. Secondly, we demonstrate further acceleration in optimization by combining FEA and Bayesian optimization. Here, we demonstrate a 42% reduction in the number of simulations necessary to find high-performing structures based employing independent BO campaigns across 5 lattice families. Moreover, we demonstrate an 82% reduction in the number of simulations necessary relative to grid-based search for $P\ge 0.90$ by combining FEA, Bayesian optimization, and one-hot encoding to find optimal lattice designs in the mixed search space. While the decrease in the number of simulations is a noteworthy reduction in the empirical load, we also highlight the reduction in the computational load from 7 days to 1.2 days for this level of performance $P$.

These results demonstrate the pivotal role that ML could play in designing lattice structures for a variety of applications where simulators for the property of interest are accessible. The integration of simulation frameworks with custom ML approaches enables an acceleration in the design optimization process without losing understanding about the impact of design variables. Collectively, the combination of simulation and ML provides a means for navigating the design space at reduced computational and empirical loads – both in exploration and prediction tasks. The development of intelligent design frameworks allows designers and engineers to take full advantage of the unparalleled design freedom that additive manufacturing presents for the design of the next generation of materials and structures.

## Methods

### Finite-element analysis (FEA) of lattice designs

To predict the Young’s modulus $E(x)$ of the lattice design structures, simulations of uniaxial quasi-static compression were performed on full-sized lattice designs using the finite element analysis (FEA) software package in the nTopology design software (Figure S1). The cylindrical design body in the simulations had a height of 20 mm with a diameter of 10 mm. In the simulations, displacement boundary conditions were applied to the top surface nodes, which were displaced 0.2 mm in the downward direction. The bottom surface nodes were subject to fixed–fixed boundary conditions where all displacements and rotations were constrained. For all simulations, the isotropic linear elastic material properties were with a Young’s modulus of 500 GPa and a Poisson’s ratio of 0.36. The material properties used corresponded to those of polylactic acid (PLA), a popular material used in additive manufacturing applications. The density of PLA used in this study was $\rho =$ 1.25 g/cm^3^. The value of the material properties used have been previously experimentally validated and were deemed appropriate for use in the FEA pipeline^40^.

To realize an automated workflow for FEA, the parametric designs were meshed using the nTopology design software. Several nTopology meshing features such as sharpening, and element constraints were used to allow for appropriate and rapid meshing of the lattice designs. The meshes were created from the implicit design body using a 0.05 mm tolerance which is 60 times smaller than our smallest design feature, which is the thickness of the unit cell walls. Using the nTopology design, we used the *Robust Tetrahedral Mesh* option, which meshes a solid domain using tetrahedral elements using custom nTopology algorithms. Furthermore, we employed the *Remesh Surface* option, which allows for automatic remeshing until a mesh that meets target constraints based on edge length, shape, and span angle are met. Furthermore, we used the *Mesh from Implicit Body* option with a quadratic geometric order for mesh elements along with edge length and regularity factor variables. We have included a table below with the parameters used in the meshing process (Table 1). Furthermore, the average computational cost of each simulation was approximately 37 min with a minimum run time of 10 min and a maximum run time of 1 h and 48 min (Figure S6). The estimated run times in the histogram were obtained using simulation time stamps to approximate the computational cost, as exact run times were not saved in the simulation output file. A total of 266 simulation times were available to calculate the estimated computational cost.

**Table 1:** Relevant parameters in the nTopology software used in the meshing of the lattice designs.

Mesh parameter	Value	Units
Mesh from implicit body		
Tolerance	0.05	mm
Remesh surface		
Edge length	1	mm
Shape	Triangle	N/A
Span angle	30	degree
Growth rate	2	N/A
Feature angle	45	degree
Mind edge length	0	mm
Robust tetrahedral mesh		
Edge length	1	mm
Regularity factor	10	N/A
Geometric order	Quadratic	N/A

## Surrogate modeling

To build the surrogate models, we used gaussian process regression (GPR) with automatic relevance determination (ARD). To train the model, we used a 75/25 training/testing split of the dataset, where the training and testing sets were randomly selected. This approach was used for building surrogate models of Young’s modulus $E(x)$ and ratio of Young’s modulus to mass $\widetilde{E}\left(x\right)$. To build a corresponding surrogate model for each target property, we used one-hot encoding to represent the five lattice families (Figure S5). In turn, this resulted in a 9-dimensional design space where five variables corresponded to the one-hot encoded vector and the remaining four corresponded to the continuous design variables ${x}_{UC}=\left(t, U{C}_{x}, U{C}_{y}, U{C}_{z}\right)$. The kernel used in the gaussian process formulation was a squared exponential kernel and was parametrized by $d+1$ parameters, where the design space dimensionality was $d=9$ due to the one-hot encoding approach. The hyperparameters of the gaussian process were initialized by dividing the range of each design variable by a factor of 10. The GPy software package (http://sheffieldml.github.io/GPy/) was used to build the gaussian process.

## Bayesian optimization formulation

Simulated learning campaigns were conducted to assess the performance $P$ of the Bayesian optimization (BO) framework to optimize $E(x)$. In the approach used to optimize $E(x)$ for individual lattice designs, 50 independent learning campaigns were conducted, where the first 10 initial simulations for each simulated campaign were selected using Latin Hypercube Sampling (LHS). The total campaign budget was set at 100 simulations. Due to the convergence observed (Fig. 5A), subsequent simulated learning campaigns were conducted with a total simulation budget of 50 simulations (Figure S4). In all the campaigns, the belief model $B\left(x\right)$ was a gaussian process with ARD, which was re-trained when new simulation new data became available. In the approach used to optimize $E(x)$ across all lattice designs, a similar approach was used to the individual lattice designs approach, except for the feature space which was represented using a one-hot encoding approach to enable optimization over a mixed-search space. To assess the performance of the BO agent across approaches and campaigns, the performance $P$ metric was defined as $P=\frac{E\left(\text{argmax}\left({B}_{i}\left(x\right)\right)\right)}{\text{max}\left(E\left(x\right)\right)}$, where ${B}_{i}\left(x\right)$ is the belief model trained after $i$ simulations. Based on the defined metric, $P=1$ indicates that the theoretical maximum has been found and values close to $P=1$ indicate high-performing designs.

### Supplementary Information

Supplementary Figures.

## Data Availability

Data is provided within the manuscript or supplementary information. Data sets generated during the current study are available from the corresponding author on reasonable request.
