# Supplementary material for: Accelerating the design of lattice structures using machine learning
Source: Sci Rep. 2024 Jun 14;14:13703. doi: 10.1038/s41598-024-63204-7 (PMC11176347; doi:10.1038/s41598-024-63204-7)
Supplement: Supplementary file 1 — Supplementary Figures. [file 41598_2024_63204_MOESM1_ESM.pdf]

Supporting Information

Accelerating the Design of Lattice Structures Using Machine Learning

Aldair E. Gongora[1]*, Caleb Friedman[1], Deirdre K. Newton[1], Timothy D. Yee[1], Zachary Doorenbos[1], Brian Giera[1], Eric B. Duoss[1], Thomas Y.-J. Han[1], Kyle Sullivan[1], Jennifer N. Rodriguez[1]

1. Lawrence Livermore National Laboratory, 700 East Avenue, Livermore, CA 94550, USA
*gongora1@llnl.gov

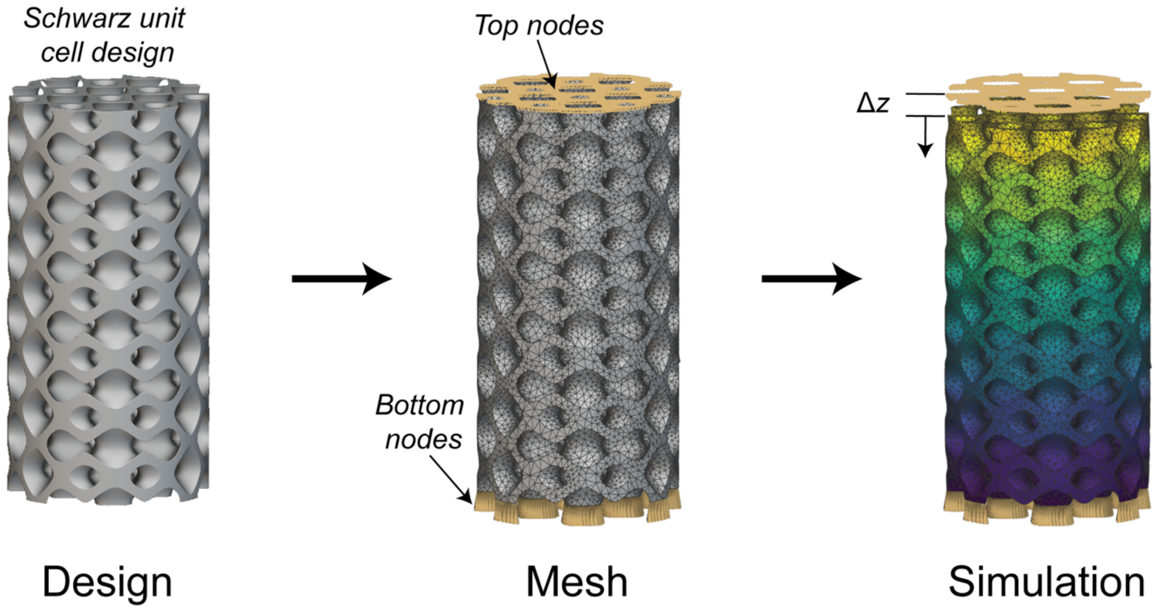

Figure S1: The parametric designs were meshed using tetrahedral mesh elements and simulations of quasi-static compression testing were conducted using finite element analysis (FEA).

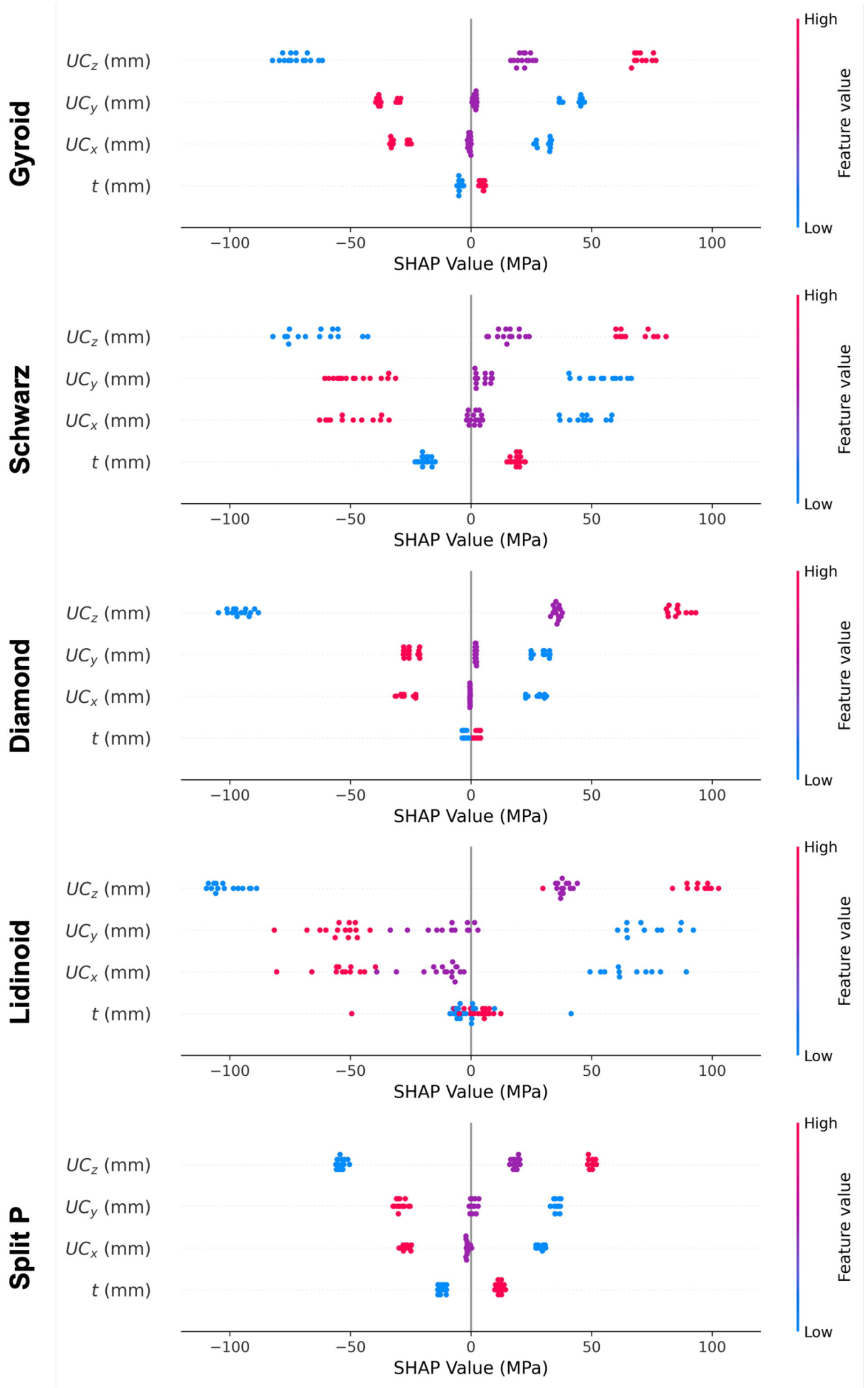

Figure S2: Shapley additive explanations (SHAP) analysis for the five lattice design values. The impact of each feature on Young's modulus $E(x)$ is depicted with each marker corresponding the SHAP value and where the color of the marker represents the relative value of the feature in the dataset. The horizontal location shows whether the effect of that feature value contributed positively or negatively in that prediction instance.

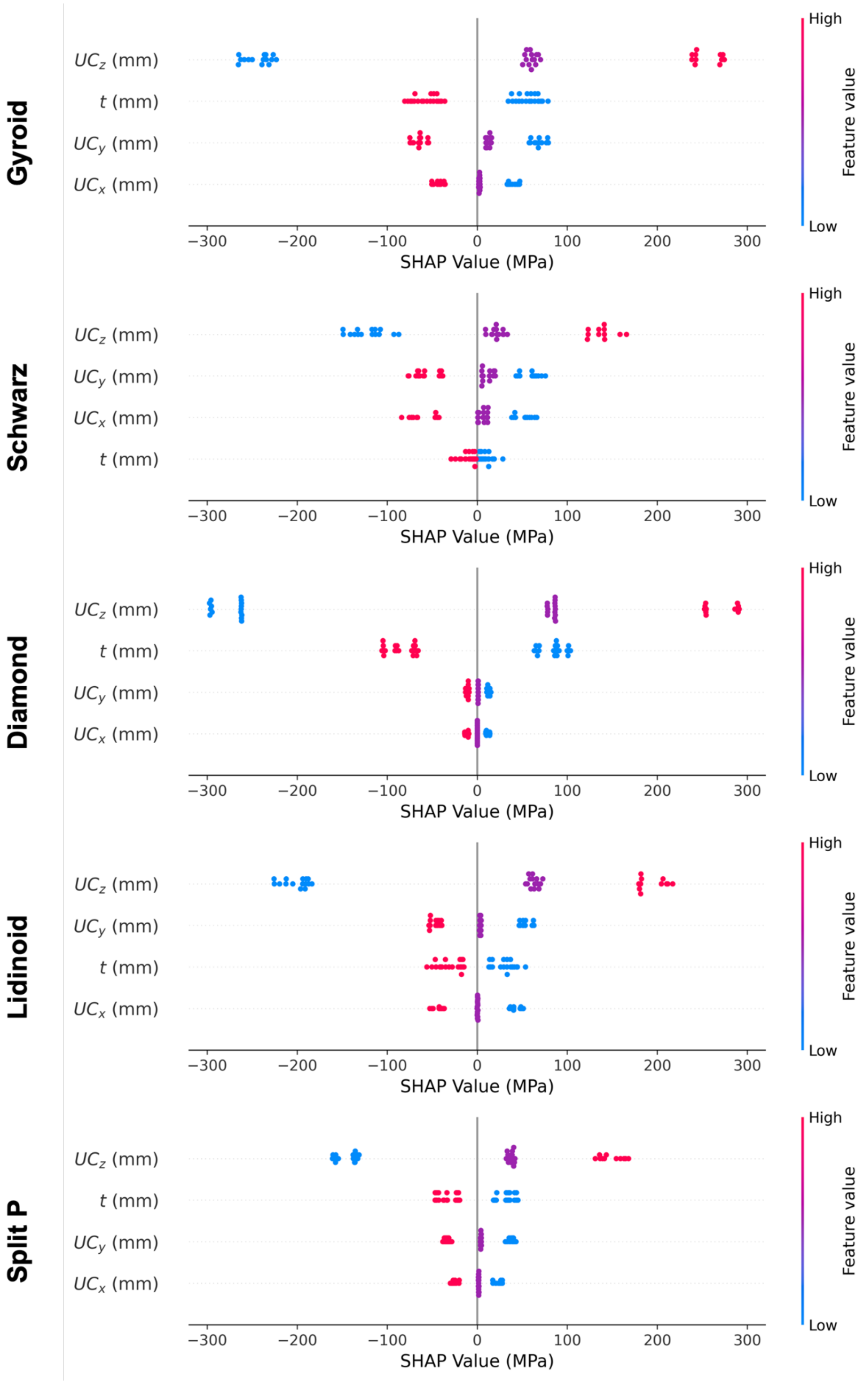

Figure S3: Shapley additive explanations (SHAP) analysis for the five lattice design values. The impact of each feature on specific Young's modulus $\widetilde{E}(x)$ is depicted with each marker corresponding the SHAP value and where the color of the marker represents the relative value of the feature in the dataset. The horizontal location shows whether the effect of that feature value contributed positively or negatively in that prediction instance.

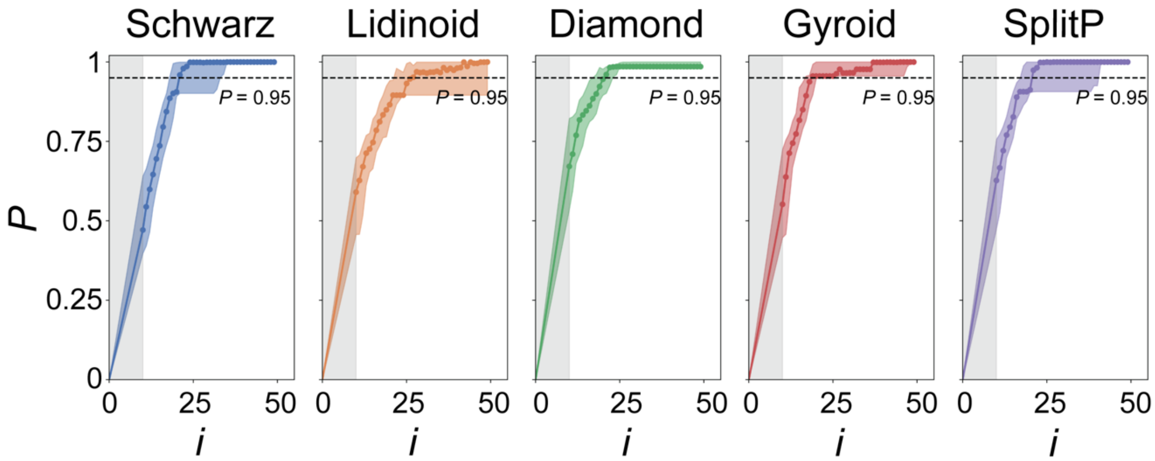

Figure S4: Independent simulated learning campaigns for each individual lattice design family. A total of 50 independent learning campaigns were conducted for each lattice design family using a Bayesian optimization (BO) approach. The performance *P* of the BO agents are plotted versus the number of simulations conducted *i*. The corresponding median performance across the 50 independent learning campaigns is depicted with the circular markers and the 25th and 75th percentiles are plotted as the lower and upper shaded regions, respectively. The ultimate performance of the BO agent was determined using a threshold of $P = 0.95$.

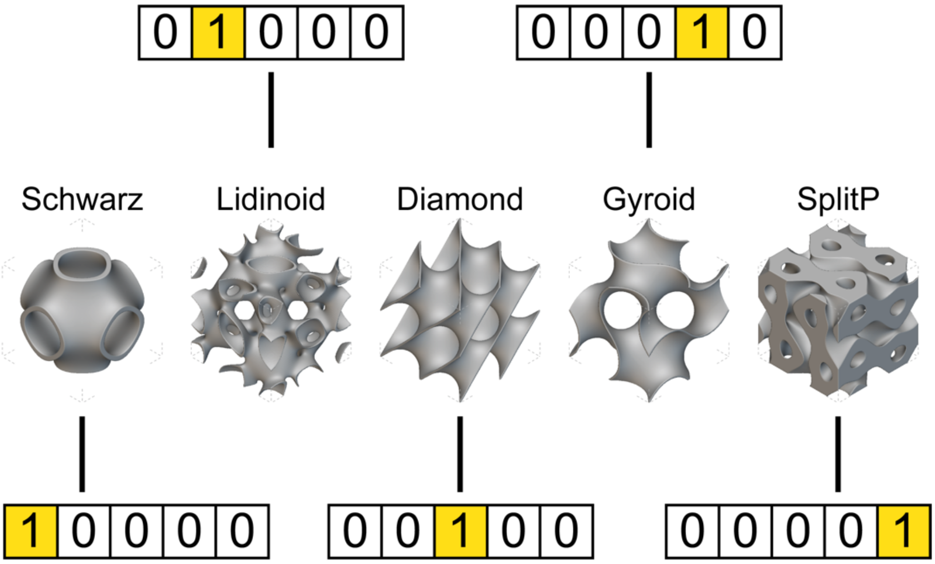

Figure S5: One-hot encoding of each lattice design family.

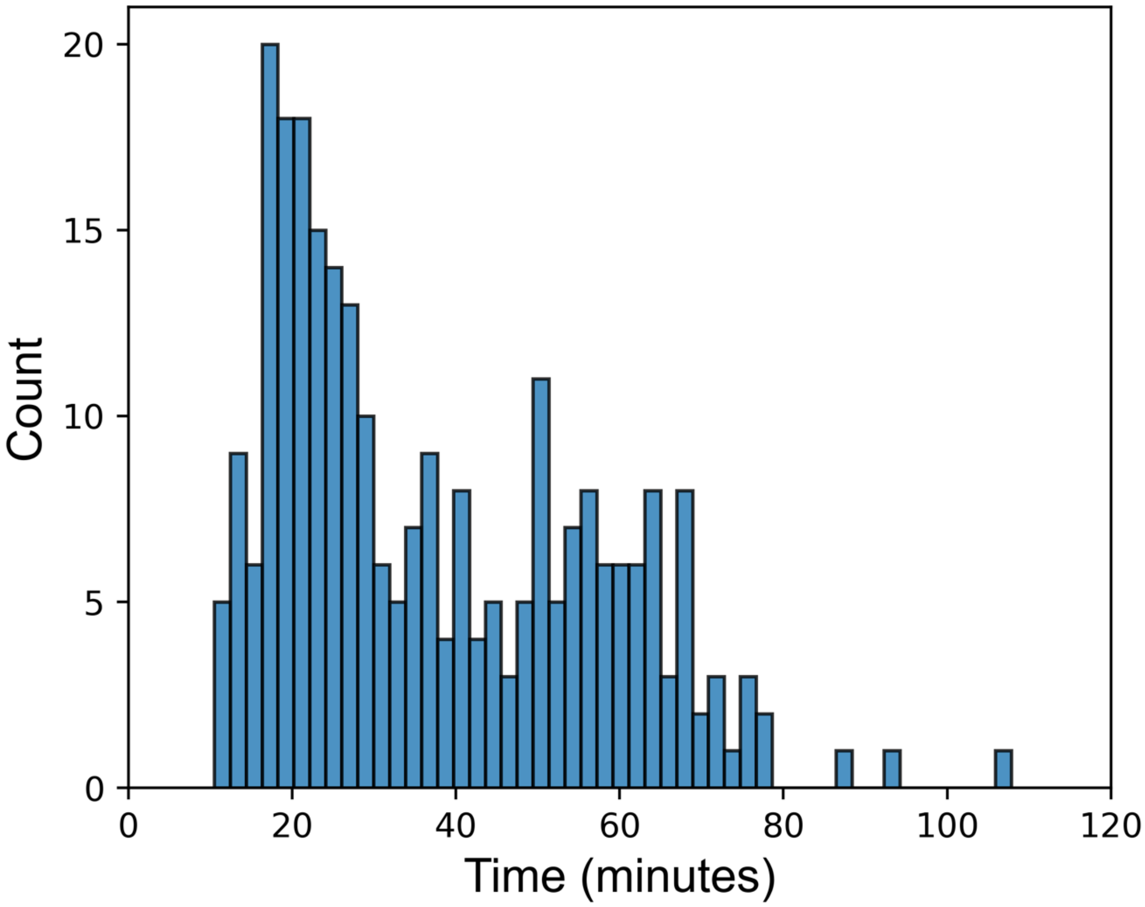

Figure S6: Histogram of estimated elapsed simulation time for simulations in the grid-search.

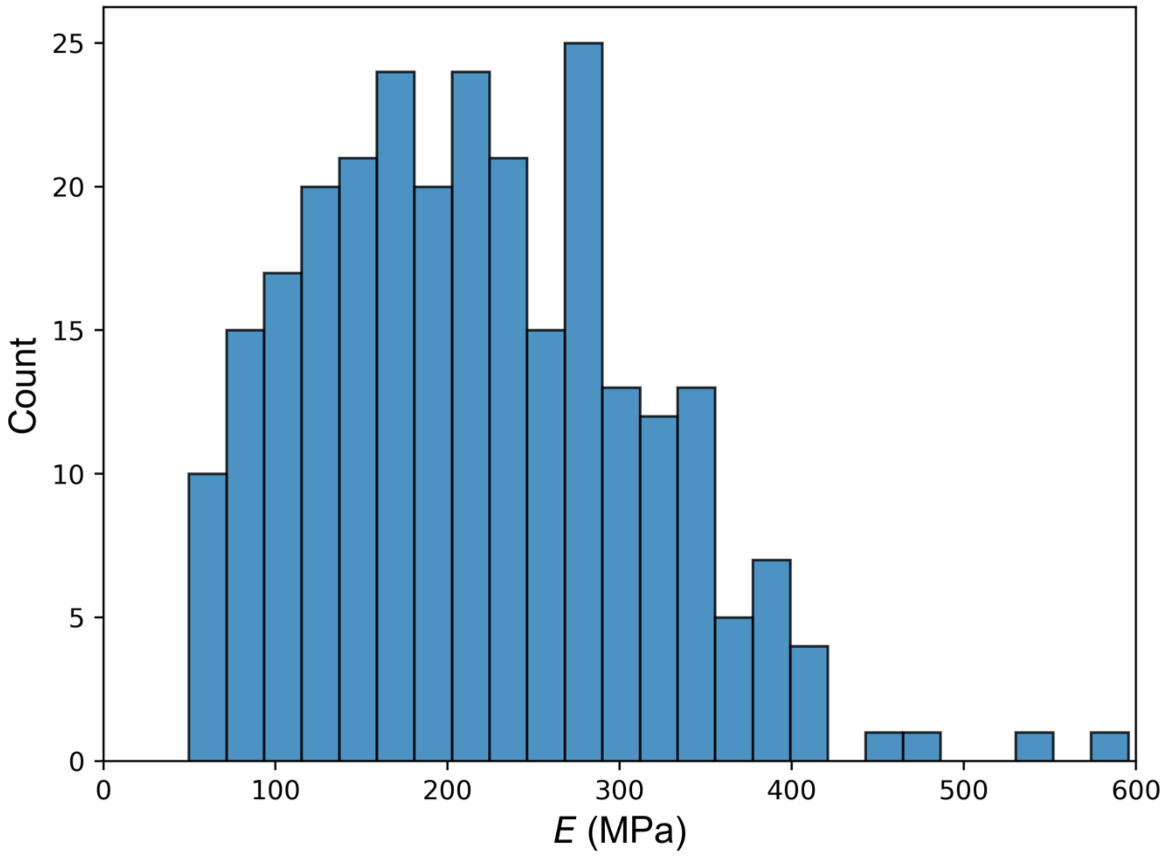

Figure S7: Histogram of *E* values from the grid-based search.

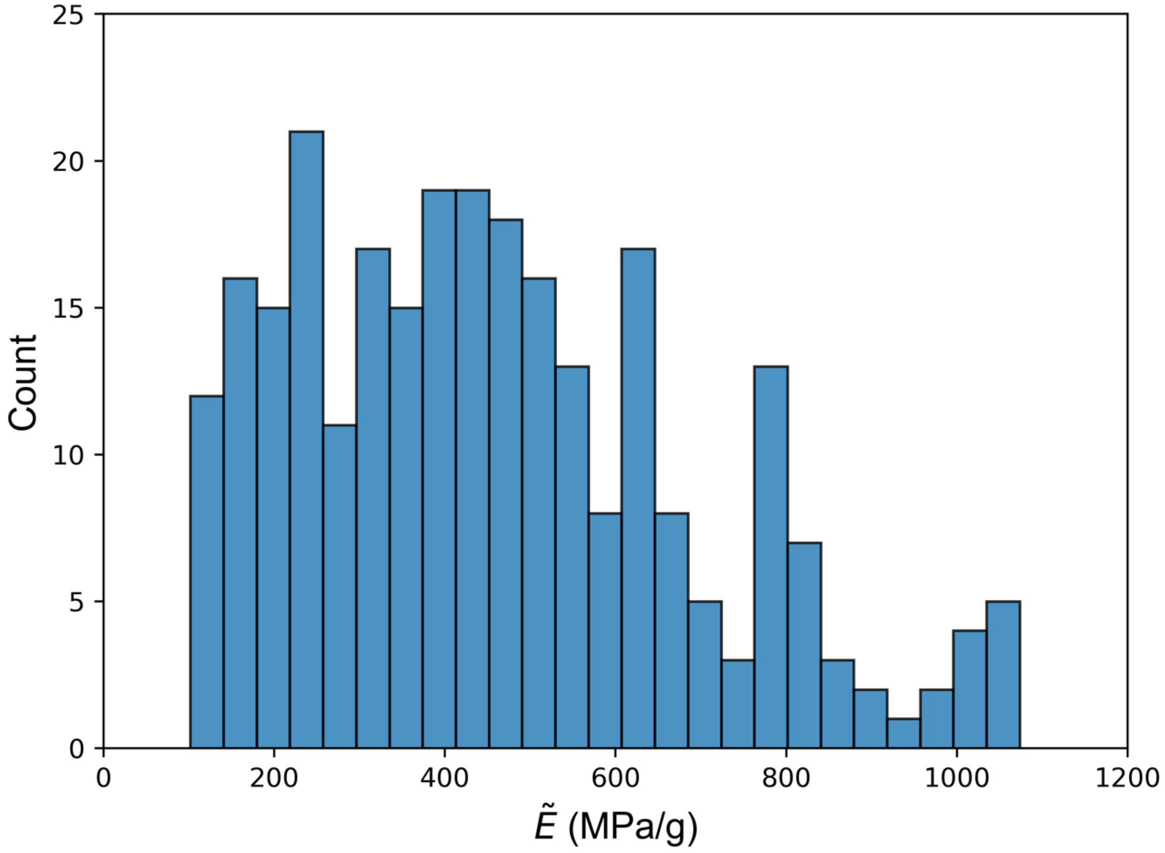

Figure S8: Histogram of $\widetilde{E}$ values from the grid-based search.
